# Clinically assessed and perceived unmet mental health needs, health care use and barriers to care for mental health problems in a Belgian general population sample

**DOI:** 10.1186/s12888-022-04094-9

**Published:** 2022-07-07

**Authors:** Eva Rens, Joris Michielsen, Geert Dom, Roy Remmen, Kris Van den Broeck

**Affiliations:** 1 Collaborative Antwerp Psychiatric Research Institute (CAPRI), University of Antwerp, Antwerp, Belgium; 2 Family Medicine and Population Health (FAMPOP), University of Antwerp, Antwerp, Belgium; 3 University of Antwerp, Gouverneur Kinsbergencentrum Room 00.56, Doornstraat 331, 2610 Wilrijk, Belgium; 4 Institute of Tropical Medicine (ITM), Antwerp, Belgium

**Keywords:** Public mental health, Mental health needs, Unmet need, Perceived need, Epidemiology, Treatment gap, Health care use, Barriers

## Abstract

**Background:**

Mental health problems often remain undetected and untreated. Prior research suggests that this is mainly due to a lack of need-perception and attitudinal barriers. The aim of this study is to examine unmet mental health needs using both a clinically assessed and a self-perceived approach in a Belgian province.

**Methods:**

A cross-sectional survey study with a weighted representative sample of 1208 individuals aged 15 – 80 years old was carried out in 2021 in the province of Antwerp (Belgium). Mental health needs were defined as a positive symptom screening for depression (PHQ-9), anxiety (GAD-7) or alcohol abuse (AUDIT-C and CAGE), combined with experiencing significant dysfunction in daily life. Also 12-month health care use for mental health problems, self-perceived unmet mental health needs and reasons for not seeking (extra) help were assessed. Logistic regression analyses were used to explore the predictors of mental health problems, health care use, and objective and subjective unmet mental health needs.

**Results:**

One in five participants had a positive screening on one of the scales, of whom half experienced dysfunction, leading to a prevalence of 10.4% mental health needs. Among those, only half used health care for their mental health, resulting in a population prevalence of 5.5% clinically assessed unmet mental health needs. Fourteen percent of the total sample perceived an unmet mental health need. However, more women and younger people perceived unmet needs, while clinically assessed unmet needs were higher among men and older people. One in six of the total sample used health care for their mental health, most of whom did not have a clinically assessed mental health need. Motivational reasons were most often endorsed for not seeking any help, while a financial barrier was the most important reason for not seeking extra help.

**Conclusions:**

The prevalence of unmet mental health needs is high. Assessed and perceived (unmet) mental health needs are both relevant and complementary, but are predicted by different factors. More research is needed on this discrepancy.

## Background

Public mental health looks into mental health problems at the population level. Besides mental health promotion and prevention of mental disorders, one of the main aims of public mental health policies is to reduce mental health inequalities [1]. Mental health inequalities in the population are often described in terms of unmet mental health needs. An unmet mental health need is present when someone has a mental health problem but does not seek or receive mental health care [2–5]. Classically, this is operationalized as having a mental disorder, as assessed with validated instruments or a diagnostic assessment, in combination with the absence of any formal health care use for mental health related reasons [2]. For example, in the European Study of the Epidemiology of Mental Disorders (ESEMeD) in the early 2000s, it was found that about half (48%) of those with a disabling 12-month mental disorder reported not using any type of formal health care and only one in four (25%) reported seeing a mental health specialist in the 12 months prior to the interview [2]. At population level, 3% of adults in Europe have an unmet mental health need. Moreover, Demyttenaere et al. (2004) estimated that at least half of serious cases receive no treatment while the majority of people in treatment are subthreshold cases [5].

A shortcoming of clinically assessed approaches (i.e., approaches using validated instruments with population norm scores to distinguish cases from non-cases) is that they do not take the subjective perception of the individual into account, while perceiving a need for mental health care is a major explanatory factor of help seeking [6–8]. Olsson et al. (2020) have therefore proposed an extended definition of unmet mental health needs, in which unmet needs can occur at three stages of the pathway to adequate care: 1) not perceiving a need for care, 2) not seeking care, and 3) not receiving (sufficient or adequate) care [9]. Applying this definition in a Swedish population sample, they found that more than one in three (36%) had perceived a need for mental health care at any time in life [9]. Among these ‘need-perceivers’, 71% sought care, and one in four care-seekers did not experience the care as sufficient [9].

In Europe, 9% percent of the general population and 33% of those with a mental disorder perceived some need for mental health care [10]. Among those with a disabling 12-month mental disorder, 82% of those with a perceived need as compared to 11% of those without a perceived need used some kind of professional help, highlighting the relevance of need perception as a major predictor of help-seeking behavior [10]. In the US, a total of 6% adults reported they felt a ‘perceived need for mental health care in the past year (2018) that was not received’ [11].

Some studies have used the more extensive Perceived Need for Care Questionnaire (PNCQ) which assesses whether mental health needs are fully met, partially met or unmet for specific healthcare services [12]. Using the PNCQ, it was found that only a minority of all mental health needs of Dutch and Australian people with a mental disorder are fully met [13]. In a Canadian general population sample, it was found that 18% reported that all their care needs were unmet, and this was especially the case for counselling needs [14].

Besides not perceiving a need for care, several other barriers to mental health care exist. Previous research has shown that attitudinal and motivational barriers, such as preferring to manage problem on one’s own, are more often reported than structural barriers such as the availability of services [4, 7, 15]. The cost of services is a particularly important barrier in the US, as compared to European countries with universal health coverage [15].

In the present cross-sectional study, the prevalence and predictors of mental health problems, assessed and self-perceived needs for mental health care as well as health care use for mental health problems and barriers to care are investigated in the province of Antwerp, Belgium. Prior explorative qualitative research in the region suggested that unmet mental health needs are high, especially among people living in poverty, ethnic minorities, and in the young and oldest age groups [16]. We use the terms clinical or assessed needs when (unmet) mental health needs are assessed using scales and clinically relevant criteria. The term subjective or perceived (unmet) needs is used for self-perceived mental health needs as reported by the individual.

## Methods

### Design and survey sample

The study is part of a research project which aims to assess the (unmet) mental health needs of the Antwerp Province in Flanders, Belgium. The study was carried out in one rural and one urban primary care zone (PCZ). PCZs are regional structures consisting of approximately 100 000 inhabitants and designed to improve the collaboration between local authorities and care providers. The rural PCZ (the region of Mol) provides a less extensive range of mental health care in the area, while the urban PCZ (the east region of Antwerp city) is highly multicultural and has a more extensive range of mental healthcare.

A sample of 5000 inhabitants aged 15 to 80 years was invited to participate in a mental health survey. The sample was randomly drawn from the national register and was stratified by gender, municipality, age and nationality (Belgian versus non-Belgian). Non-Belgians were oversampled as a lower response rate was expected in this group, based on previous research. Invited individuals received two postal invitations between May and July 2021. The first letter included a link and QR-code to the online questionnaire, and the second invitation also included a Dutch paper questionnaire that could be returned free of charge. The online questionnaire was available in six languages: Dutch, French, English, German, Polish and Arabic. The questionnaire consisted of 94 questions in total, but the vast majority of participants did not have to answer all questions due to skip logic. Forced response was implemented in the online questionnaire to avoid missing data, but participants could still indicate ‘I don’t know’ or ‘not applicable’ on most questions. The completion time was estimated at ten minutes. Informed consent was obtained from all subjects and/or their legal guardian. All methods were carried out in accordance with relevant guidelines and regulations.

### Variables and instruments

The questionnaire was fully self-report and consisted of socio-demographic questions, screening scales for common mental health problems, and questions about perceived unmet mental health needs. Depending on the given answers, additional questions about the number and type of professional health care providers which were consulted for mental health problems or the barriers to care were presented.

The following socio-demographic information was included in the current study: age category (15 – 25y old, 26 – 39y old, 40 – 64y old, 65 – 80y old), gender (M, F), origin (geographic region of Europe, non-Europe), educational attainment (primary education, secondary education, higher education i.e. college or university), financial distress (self-reported financial difficulties or not) and urbanicity of the residence (urban, rural).

#### Mental health problems

Short screening questionnaires were used for depression, anxiety disorder and alcohol disorder. The presence of depression was assessed using the Patient Health Questionnaire-9 (PHQ-9 [17]) and the diagnostic DSM-IV algorithm was used to distinguish people with and without any type of probable depressive disorder (both Major Depressive and Other Depressive Syndrome). This scoring method was chosen as it yields a higher specificity, but the ‘other depressive syndrome’ scoring (positive screening when at least two symptoms are indicated at least at more than half the days and one of the symptoms is depressed mood or anhedonia) was included as well to compensate for lower sensitivity [18].

The presence of clinical anxiety was assessed using the Generalized Anxiety Disorder-7 (GAD-7 [19]), which has good validity and reliability for screening in the general population [20]. A score of 10 or higher indicates the presence of a probable generalized anxiety disorder.

Two short questionnaires were used to assess alcohol abuse. The AUDIT-C assesses frequencies and quantities of alcohol consumption, and a cut-off point of ≥ 5 for men and ≥ 4 for women was used [21]. The four CAGE questions examined alcoholism and a score of ≥ 2 was used as a cut-off point [22]. The presence of an alcohol disorder was defined as a positive screening on both the AUDIT-C and the CAGE.

Next, dysfunction in daily life due to psychological problems was examined using a short version of the Sheehan disability scale [23]. All respondents indicated the extent to which their social and leisure life, work or study, and family life were affected by psychological problems on a scale from zero to ten. A score of at least six on one of the scales reflects moderate dysfunction and was considered a significant level of dysfunction.

A ‘clinical’ mental health problem is then defined as the presence of at least one positive screening on one of the mental health scales in combination with the presence of significant dysfunction.

#### Health care use for mental health problems

All participants answering ‘yes’ on the question “In the past 12 months, have you been in contact with a professional care provider (e.g., general practitioner, psychologist,…) because of psychological problems, your emotions, or alcohol or drug use?” were asked to specify whether they had contact with a general practitioner, a psychologist or psychotherapist, a psychiatrist, and whether they were prescribed medication for mental health problems.

#### Clinically assessed unmet mental health needs

A clinically assessed unmet mental health need is defined as the presence of any clinical mental health need together with the absence of health care use for mental health problems in the past 12 months. No distinction was made between the type of care provider or the number of contacts.

#### Perceived unmet mental health needs and barriers

Participants without any twelve-month professional contact for mental health problems were asked “During the past 12 months, have you thought you might need help for psychological problems, your emotional problems, or alcohol or drug use?”. Those replying ‘yes’, are considered those with ‘perceived fully unmet needs’, and those replying ‘no’ as those with ‘no perceived need’. On the other hand, participants reporting health care use for mental health problems in the past twelve months were asked whether they thought the received care was sufficient. Respondents replying with ‘yes’, are considered as those with ‘met needs’, whereas those replying with ‘no’ are considered as those with ‘perceived partially unmet needs’.

All subjects who responded that they thought they might have needed help but did not seek it, or that they did not receive sufficient help, were asked to endorse all the reasons that were applicable from a list of nine reasons. This list was self-construed and based on common barriers reported in other studies [6, 7].

### Statistical analyses

The individual observations were weighed using inverse probability weighting to correct for differences in response rate between age categories, gender and nationality. Missing data were very low (< 1% for every variable) because of the forced response implementation in the online questionnaire, and therefore it was decided to use available case analysis. Participants who returned the paper questionnaire and had a high level of missing data or missing demographic information were excluded from the analysis (*n* = 14). All analyses were conducted in IBM SPSS Statistics version 28.

Descriptive characteristics of the sample and the prevalence of mental health needs, health care use for mental health problems, perceived and assessed unmet mental health needs, and barriers to care are reported using weighted percentages.

Logistic regression analyses were carried out to assess the likelihood of having a mental health problem, using health services for mental health problems, having an assessed unmet mental health need, and having a perceived unmet mental health need. The logistic regression models of the presence of a mental health problem, health care use and perceived unmet needs consider the full sample, while the logistic regression modelling assessed unmet needs considers only those with any mental health problem. All multivariable models include all sociodemographic factors. The multivariable models of health care use and perceived unmet needs also include the presence of any assessed mental health need.

Unadjusted odds ratios (UOR) and adjusted odds ratios (AOR) are reported with their 95% confidence intervals (CI). *P*-values of the comparisons to the reference and the overall significance of the factor in the multivariable model are indicated by asterisks. Interactions were not included. A significance level of *p* < 0.05 was established for all analyses.

## Results

### Participants

A total of 1208 people (24.2% response rate) fully participated, most of them (79.2%) online. The vast majority (93.5%) completed the questionnaire in Dutch, 3.2% in English, 1.4% in French, 1.3% in Arabic and 0.5% in Polish. Non-response was higher among men, younger people and people with a birthplace outside Europe.

In the weighted sample, 49.8% were women. The mean age was 45.5 years old (SD = 17.8), with 16.5% people aged 25 or younger, 32.4% aged between 26 and 44 years old, 33.7% aged between 45 and 64 years old and 17.3% aged 65 or older. One in nine (11.1%) participants was born outside of Europe, and 55.6% lived in an urban residence. As regards education, 13.9% did not have a secondary education degree and 41.1% has a higher education degree. More than one in six (17.5%) reported financial distress in the past twelve months.

### Prevalence and predictors of mental health problems

Approximately one in five (21.5%) has a positive screening on one or more of the mental health scales: 10.2% had a possible depressive disorder, 10.0% a possible anxiety disorder, and 8.8% a possible alcohol disorder. A total of 20.3% of the sample experiences significant dysfunction in daily life due to psychological problems, but this does not completely overlap with the group of people with a mental health need as assessed by the screening questionnaires. Approximately half (49.6%) of those with a positive screening experienced dysfunction in daily life due to their mental health, as opposed to 12.4% of those without a positive screening. A clinical mental health problem (i.e., the presence of both a positive screening and dysfunction) is then present in 10.4% of the sample.

Logistic regression analysis was used to assess the likelihood of having a clinical mental health problem (Table 1). There was no significant gender effect. Age is a significant predictor of having any mental health need, such that younger age groups have a higher prevalence of mental health problems as compared to older people. Specifically, compared to people aged 25 or less, mental health problems were less common among people between the ages of 45 and 64 (OR = 0.37, 95% CI = 0.21—0.66) and people aged 65 or older (OR = 0.17, 95% CI = 0.07—0.40). Compared to people with a primary education degree, the likelihood of having a mental health problem was lower among people with a secondary education degree (OR = 0.56, 95% CI = 0.33—0.95) or a higher education degree (OR = 0.50, 95% CI = 0.27—0.91). Financial distress was strongly predictive of mental health problems (OR = 3.67, 95% CI = 2.35 – 5.72). Finally, mental health problems were more common in people living in an urban residence, but this effect was not significant when other factors were taken into account.

**Table 1:** Logistic regression analysis modelling the likelihood of having a clinical mental health problem in the general population (*N* = 1208)

			Clinical mental health problem			
		%	UOR	UOR 95% CI	AOR	AOR 95% CI
Gender	Male (ref.)	10.1				
	Female	10.8	1.08	.75—1.57	.1.22	.82—1.80
Age***	15–25 (ref.)	18.7				
	26–44	13.2	.66	.42—1.05	.78	.45—1.34
	45–64	7.2	.34***	.20—.57	.37***	.21—.66
	65–80	3.5	.16***	.07—.36	.17***	.07—.40
Education	Primary (ref.)	20.6				
	Secondary	9.5	.40***	.25—.65	.56*	.33—.95
	Higher	8.1	.34***	.21—.56	.50*	.27—.91
Financial distress***	No (ref)	7.7				
	Yes	23.4	3.67***	2.47—5.44	3.67***	2.35—5.72
Birthplace	Europe (ref.)	9.8				
	Non-Europe	15.3	1.65	.99—2.76	.74	.41—1.32
Urbanicity	Urban (ref.)	12.6				
	Rural	7.7	.58**	.39—.86	.67	.44—1.01

*UOR* Unadjusted Odds Ratio

*AOR* Adjusted Odds Ratio (adjusted for all variables)

^*^p < .05 **p < .01 ***p < .001

Asterisks after the variable name represent significance of the factor in the multivariable model

### Prevalence and predictors of health care use for mental health problems

Considering the total sample, one in six (17.6%) reported health care use for mental health in the past twelve months. Psychologists (11.4%) and GPs (11.3%) were more often consulted compared to psychiatrists (3.6%), and 6.4% was prescribed medication for mental health problems. Results of the logistic regression analysis examining the predictors of health care use for mental health problems in the general population are shown in Table 2.

**Table 2:** Logistic regression analysis modelling the likelihood of using health services for mental health in the general population (*N* = 1208)

			Health service use for mental health			
		%	UOR	UOR 95% CI	AOR	AOR 95% CI
Gender***	Male (ref.)	12.9				
	Female	21.4	1.84***	1.35—2.50	1.91***	1.37—2.66
Age**	15–25 (ref.)	16.5				
	26–44	23.1	1.52	.97—2.36	1.37	.81—2.32
	45–64	17.6	1.08	.69—1.69	1.14	.68—1.92
	65–80	5.7	.31***	.15—.61	.31**	.15—.65
**Education**	Primary (ref.)	14.1				
	Secondary	15.9	1.16	.71—1.90	1.47	.82—2.61
	Higher	19.4	1.47	.90—2.41	1.75	.95—3.22
Financial distress**	No (ref)	15.4				
	Yes	25.7	1.91***	1.34—2.72	1.81**	1.19 – 2.75
Birthplace**	Europe (ref.)	17.6				
	Non-Europe	13.4	.73	.43—1.22	.41**	.22—.75
**Urbanicity**	Urban (ref.)	18.9				
	Rural	14.9	.75	.55—1.02	.80	.57—1.12
Mental health need***	No (ref)	13.6				
	Yes	47.6	5.74***	3.88 – 8.48	5.31***	3.44 – 8.19

*UOR* Unadjusted Odds Ratio

*AOR* Adjusted Odds Ratio (adjusted for all variables)

^*^p < .05 **p < .01 ***p < .001

Asterisks after the variable name represent significance of the factor in the multivariable model

A significant difference in the likelihood of using health services for mental health problems was found, with 21.4% women as compared to 12.9% men consulting a health professional for their mental health (OR = 1.91, 95% CI = 1.37 – 2.66). Age is a significant predictor of health care use, with people aged 65 and older being less likely to consult a professional for mental health problems compared to the reference of 15 – 25-year olds (OR = 0.31, 95% CI = 0.15—0.65). Origin and financial distress are significant predictors well. Individuals with financial distress were significantly more likely to consult a health care professional for mental health problems (OR = 1.81, 95% CI = 1.19 – 2.75). Individuals with a non-European origin were less likely to use health services for their mental health in the multivariable model only (OR = 0.41, 95% CI = 0.22—0.75).

Besides the sociodemographic predictors, also the presence of a clinical mental health problem was included. As expected, this was highly predictive of using health care for mental health problems, with 13.6% of those without as compared to 47.6% of those with a mental health problem using health care for their mental health (OR = 5.31, 95% CI = 3.44 – 8.19).

### Prevalence and predictors of clinically assessed unmet mental health needs

**Fig. 1:**
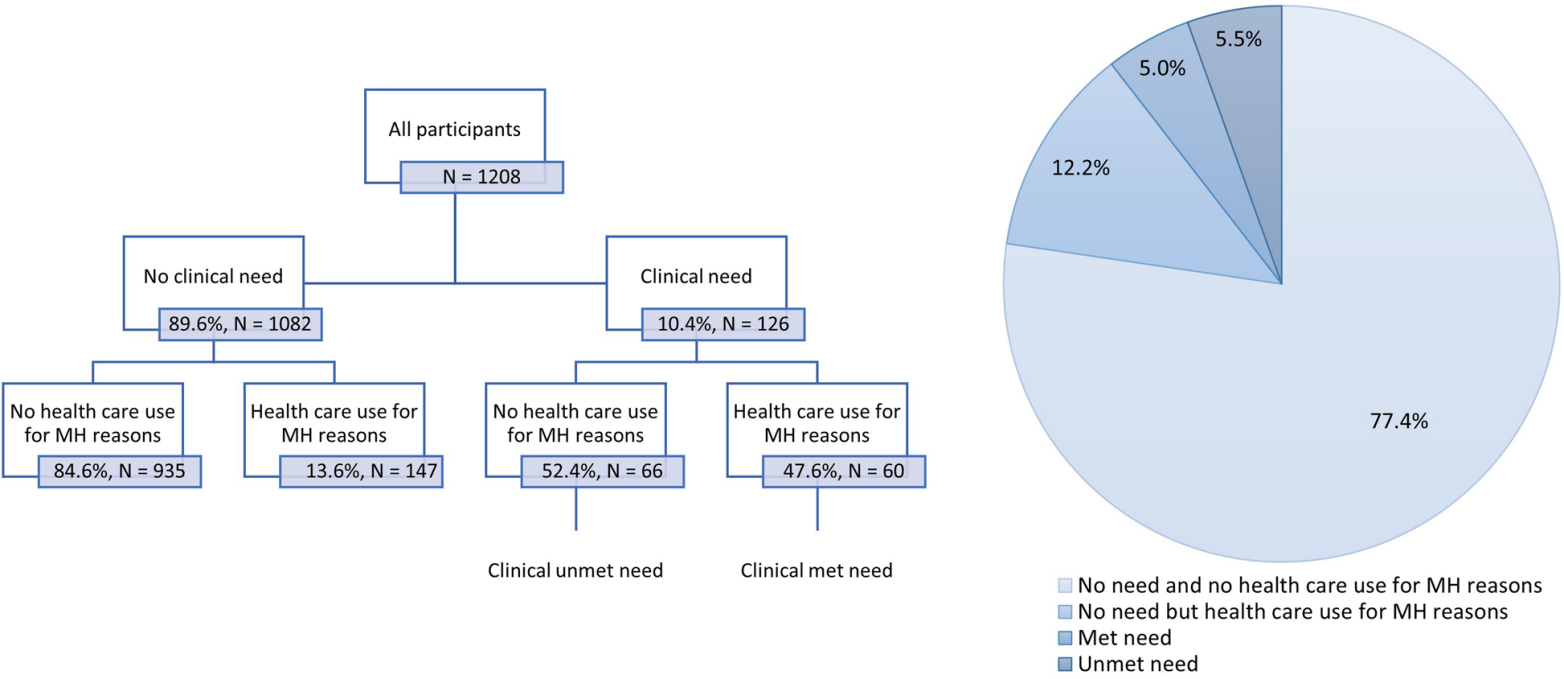
Classification and population distribution of clinical (unmet) needs for mental health care

The classification and population distribution of assessed unmet mental health needs is shown in Fig. 1. In the total sample, 5.5% presents a clinical unmet need for mental health care. Moreover, only 29.0% of all people who used health services for mental health reasons has a clinical mental health problem.

Table 3 shows the distribution and logistic regression analysis of having a clinical unmet mental health need. Note that both the percentage within the category as a whole is shown, as well as the percentage within the category with a mental health need, as the former is highly dependent on the prevalence of mental health problems in that group. The logistic regression results therefore apply to the subsample of people with a mental health problem (*N* = 126). Due to the lower sample size and low incidence of unmet need in some subgroups, wide confidence intervals arise and the findings should be interpreted with caution. An estimated 6.8% of all men have a clinical unmet need as compared to 4.2% of all women. Among women with a mental health need, 38.6% did not use health services, which is significantly less than the 67.6% men with a mental health need (OR = 0.20, 95% CI = 0.08—0.49). Age is not a significant predictor. However, as the prevalence of mental health problems among individuals aged 65 or older is very low (*N* = 7), and none of them used health services for their mental health, this leads to a 100% prevalence of unmet need in this subgroup. Individuals with a mental health need experiencing financial distress are significantly less likely to have an unmet mental health need as compared to those without financial distress (OR = 0.22, 95% CI = 0.08—0.57). Those with a non-European birthplace had a higher likelihood of having an unmet mental health need, but only in the multivariable model (OR = 3.96, 95% CI = 1.13 – 13.89).

**Table 3:** Logistic regression analysis modelling the likelihood of having a clinical unmet need, i.e., not using health care for mental health among participants with a mental health problem (*N* = 126)

				Clinical unmet need			
		total %	%	UOR	UOR 95% CI	AOR	AOR 95% CI
Gender***	Male (ref.)	6.8	67.6				
	Female	4.2	38.6	.30**	.15—.63	.20***	.08—.49
Age	15–25 (ref.)	10.5	56.2				
	26–44	6.1	46.2	.67	.29—1.56	1.14	.36—3.64
	45–64	3.4	47.0	.69	.26—1.82	.97	.30—3.12
	65–80	3.5	100.0	-	-	-	-
Education	Primary (ref.)	11.1	54.1				
	Secondary	5.4	57.4	1.14	.48—2.73	1.19	.44—3.42
	Higher	3.8	46.2	.73	.29—1.82	.50	.14—1.81
Financial distress**	No (ref)	4.8	62.7				
	Yes	8.6	36.8	0.35**	.17—.73	.22**	.08—.57
Birthplace*	Europe (ref.)	9.8	52.0				
	Non-Europe	15.3	55.1	1.13	.44—2.93	3.96*	1.13 – 13.89
Urbanicity	Urban (ref.)	12.6	52.0				
	Rural	7.7	53.5	1.06	.50—2.24	1.24	.47—3.28

*UOR* Unadjusted Odds Ratio

*AOR* Adjusted Odds Ratio (adjusted for all variables)

^*^p < .05 **p < .01 ***p < .001

Asterisks after the variable name represent significance of the factor in the multivariable model

### Prevalence and predictors of perceived unmet mental health needs

**Fig. 2:**
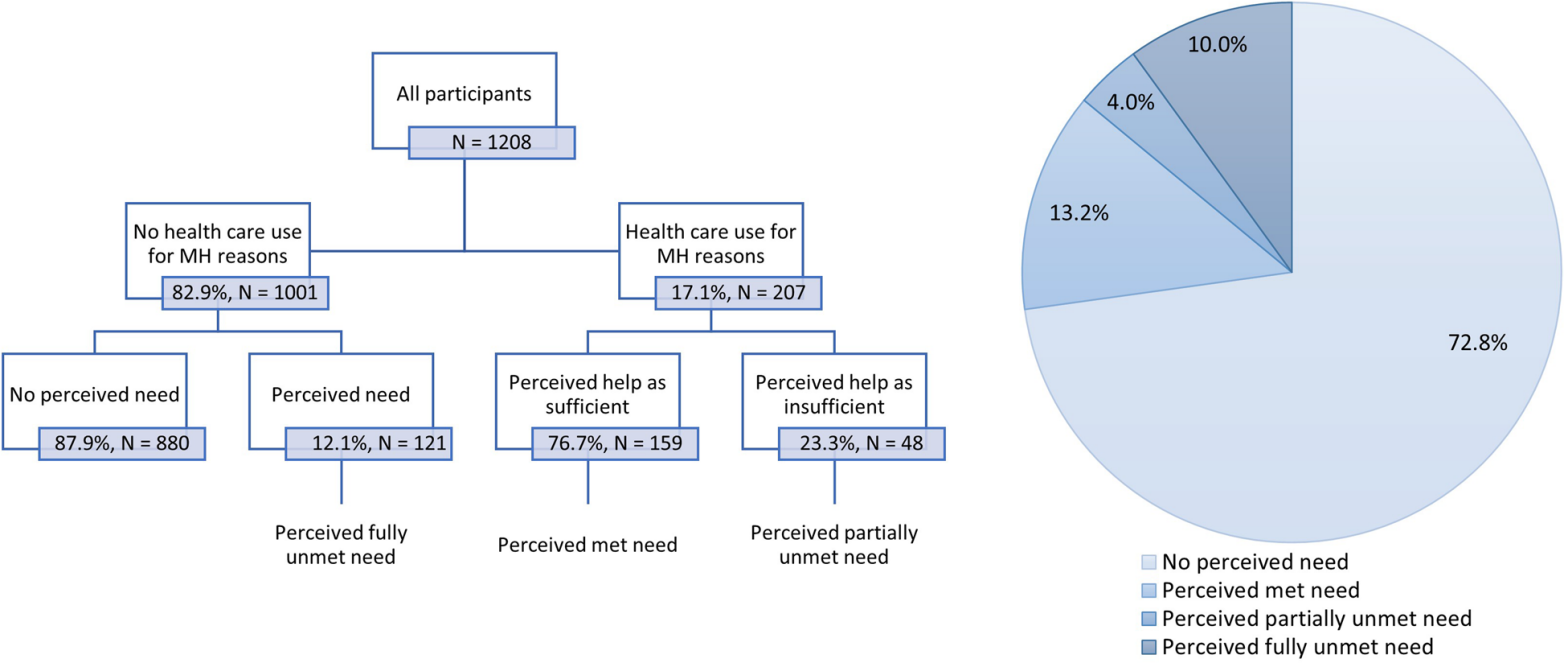
Classification and population distribution of perceived (unmet) needs for mental health care

Figure 2 shows the classification table and population distribution of perceived unmet needs. A minority of 12.1% non-care-seekers indicated that they felt a need for mental health care but did not seek help. In the general population, this translates to one in ten (10.0%) ‘perceived fully unmet needs’. Among the care-seekers, the majority (76.6%) thought the help was sufficient, resulting in 4.0% ‘perceived partially unmet needs’ in the population. Altogether, 14.0% of the population perceives an unmet mental health need.

Predictors of perceiving an unmet need for mental health care (both partially and fully) were assessed using logistic regression analysis, shown in Table 4. Women were more likely to perceive an unmet need for mental health care (OR = 1.73, 85% CI = 1.20 – 2.50). Age was a significant predictor of perceived unmet needs, as there is a trend towards more perceived unmet needs in the youngest age groups as compared to the oldest age groups. For example, 20.7% of the 26 – 44-year-olds perceived an unmet mental health need, in contrast to 11.0% of 45 – 64-year-olds and 2.8% of participants aged 65 and older. In the multivariable model, individuals aged 65 and older are significantly less likely to perceive an unmet mental health need as compared to the youngest age group (OR = 0.14, 95% CI = 0.05—0.37). Individuals experiencing financial distress have a higher likelihood to perceive an unmet mental health need (OR = 2.06, 95% CI = 1.31 – 3.25). Individuals born outside Europe are significantly less likely to perceive an unmet mental health need (OR = 0.51, 95% CI = 0.28—0.95).

**Table 4:** Logistic regression analysis modelling the likelihood of perceiving a fully or partially unmet need for mental health care in the general population (*N* = 1208)

			Any perceived unmet need			
		%	UOR	UOR 95% CI	AOR	AOR 95% CI
Gender**	Male (ref.)	11.2				
	Female	16.8	1.61**	1.15—2.24	1.73**	1.20—2.50
Age***	15–25 (ref.)	18.8				
	26–44	20.7	1.13	.73—1.74	1.18	.69—2.02
	45–64	11.0	.54**	.33—.86	.61	.35—1.06
	65–80	2.8	.13***	.05—.31	.14***	.05—.37
Education	Primary (ref.)	16.5				
	Secondary	11.6	.66	.41—1.08	.95	.53—1.71
	Higher	15.8	.95	.59—1.53	1.28	.69—2.39
Financial distress**	No (ref)	12.0				
	Yes	22.9	2.17***	1.50—3.16	2.06**	1.31—3.25
Birthplace*	Europe (ref.)	14.0				
	Non-Europe	13.7	.98	.58—1.64	.51*	.28—.95
Urbanicity	Urban (ref.)	15.4				
	Rural	12.2	.76	.55—1.06	.87	.60—1.27
Mental health need***	No	10.4				
	Yes	46.0	7.30***	4.87—10.91	5.92***	3.81—9.20

*UOR* Unadjusted Odds Ratio

*AOR* Adjusted Odds Ratio (adjusted for all variables)

^*^p < .05 **p < .01 ***p < .001

Asterisks after the variable name represent significance of the factor in the multivariable model

The presence of a clinical mental health problem was taken into account as well. Approximately one in ten (10.4%) individuals without a mental health problem perceived an unmet mental health need, compared to 46.0% individuals with a mental health problem, making the presence of a mental health need highly predictive of perceived unmet needs (OR = 5.92, 95% CI = 3.81 – 9.20).

### Barriers to mental health care

Participants who perceived an unmet mental health need were asked to indicate the reasons why they did not seek or receive (sufficient) help. The prevalence of the reported barriers to mental health care is shown in Table 5. For those who did not seek care, the most often cited reason is that they prefer to handle problems on their own (65.6%), followed by thinking it wouldn’t help (30.1%) and time constraints (27.5%). One in four (24.5%) reported the cost as a barrier. For those with partially unmet needs, cost was the most often cited barrier (43.0%), followed by a preference to handle problems on their own (38.6%). Both among those with fully unmet needs and those with partially unmet needs, about one in five people reported other barriers, for example, an expected waiting time and bad experiences in the past.

**Table 5:** Prevalence of endorsed reasons for not seeking or receiving (extra) help among participants who perceived an unmet need for mental health care

Reason	Fully unmet need (*N* = 121)	Partially unmet need (*N* = 48)
I prefer to handle problems on my own	65.6%	38.6%
I don’t think it would help	30.1%	24.1%
I don’t have time for it	27.5%	9.0%
I worry about the costs	24.5%	43.0%
I don’t know where to go for (extra) help	21.8%	26.3%
I’m afraid others would think bad of me	10.7%	22.5%
I don’t speak the language well	6.7%	7.1%
I asked for it, but didn’t get (extra) help	2.3%	16.0%
I cannot get there (e.g. no transport)	1.9%	4.8%
Another reason	20.4%	20.1%

## Discussion

This cross-sectional survey study evaluated common mental health needs (depression, anxiety and alcohol problems) in a representative general population sample in Antwerp, Belgium. A total of 1208 people aged 15 to 80 years old participated in the study. It was found that about one in five (21.5%) has a positive symptom screening for depression, anxiety and/or alcohol disorder. Half of them also experience functional problems in their daily lives because of mental health problems. Surprisingly, also 12% of those without a positive screening on one of the screening scales indicate that their daily life is at least moderately impacted by psychological problems. A clinical mental health need was defined as the presence of both a positive symptom screening and dysfunction, and was present in one-tenth (10.4%) of the sample. Mental health problems were found to be more common among younger age groups, people with a lower education level and people with financial problems.

Furthermore, only half of the people with a clinical mental health problem consulted a health care professional for their mental health, resulting in a population prevalence of 5.5% clinical unmet mental health needs. In addition, 14.0% of the population perceived an unmet mental need themselves, although the predictors of assessed and perceived unmet mental health needs differ. Because the quantity and quality of care were not taken into account, this approach may even lead to an underestimation of the actual unmet mental health needs. In prior research it was suggested that approximately half of treatments in high-income countries do not meet minimally adequate treatment (MAT) criteria (i.e., eight or more psychotherapy visits or four or more visits to a doctor with pharmacotherapy) [24–26].

Overall, one in six people discussed mental health related issues with a health care professional within the past year, especially with a psychologist and/or GP. Men, people aged 65 and older, and people born outside of Europe were less likely to use health care for their mental health. Health care use for mental health problems in the general population is higher than generally reported in other studies, where approximately one in ten people use formal health services for their mental health [27–29]. This may be due to the broader definition of health care use, namely any contact with a health professional for mental health reasons (incl. emotional problems or substance abuse).

Clinically assessed unmet mental health needs are on a population level more common among young people, but this can be explained by their higher level of mental health problems. In contrast, only 3.5% of people aged 65 and older have a mental health problem according to screening scales, but none of these participants received any form of care for mental health problems. Consistent with previous research, older people with a mental disorder are less likely to seek help when needed, especially because they tend to underestimate their own needs [30–33]. Also a remarkable gender effect is present, with 67.6% of men versus 38.6% of women with a clinical mental health problem who did not seek help.

The population share of people with financial problems with unmet mental health needs was higher than the population proportion of people without financial problems with unmet mental health needs because of their higher prevalence of mental health problems. However, individuals with financial distress with a mental health problem more often sought help. In line with this finding, a longitudinal study in the UK reported higher levels of treatment with medication and psychological therapy among people from disadvantaged backgrounds [34]. Other studies reported an increased risk of unmet needs among people with lower income [4, 35], or reported no clear association [36, 37]. Firstly, it must be noted that financial distress was self-reported in this study, and people might differ in the way they define financial difficulties. Secondly, people with more financial resources might have more possibilities for self-care, a larger informal support network or other alternatives such that professional help is less needed. Finally, the design doesn’t allow to draw causal conclusions, and the interpretation is especially difficult because of the reciprocal relationship between mental illness and poverty [38].

Another remarkable finding is that almost two thirds (71.0%) of those who discussed mental health related problems with a professional had no current mental health need as assessed by screening questionnaires. Several reasons can account for this finding. First, these individuals may be subthreshold cases, or may experience little dysfunction in daily life, or may have a different mental health problem than those assessed in the study. Second, mental health needs were assessed at point-prevalence, while health care contacts for mental health reasons were surveyed at 12-month prevalence. It may therefore be possible that some people have had a mental health problem that is already resolved. Finally, this may also be an expression of ‘overmet need’. Research has shown that people without a mental disorder account for a significant proportion of healthcare users, but that these individuals often have other need indicators, and generally have fewer visits and use less specialist services [5, 27, 39]. People with mental distress receiving some professional help should therefore not be regarded as having ‘overmet need’, as this can alleviate mild mental health problems and prevent problems from worsening.

Unmet mental health needs were also assessed from a subjective perspective. A perceived unmet mental health need is present when someone did not seek care but perceived a need for mental health care (= fully perceived unmet need), or when someone did seek care but felt that this was not sufficient (= partially perceived unmet need). In total, 14.0% perceived an unmet mental health need, of which the majority are fully unmet. When help was received, 23% felt that they were insufficiently helped. In line with previous research, men and older people were less likely to perceive an unmet need for mental health care [9, 14, 40]. Contrary to assessed unmet needs, individuals experiencing financial distress more often perceived an unmet mental health need, but this can be attributed to the different sample studied (subsample with mental health problem vs. total sample).

When an unmet mental health need was perceived, participants were asked to endorse all reasons for not seeking (extra) help. As expected from the literature, the most frequently reported barriers for not getting help are motivational or attitudinal barriers [4, 15]. Two-thirds cited self-reliance as the reason for not seeking help, and nearly a third thought it wouldn’t help. A quarter of the people who did not seek help mentioned cost as a barrier. However, among individuals who received help but felt this was insufficient, financial reasons were most often endorsed. This suggests that the cost of mental health care in Belgium is primarily an obstacle in obtaining adequate care as long as needed (e.g., the majority of psychotherapy was not reimbursed at the time of data-collection). Importantly, the questions about barriers to care were only asked to need-perceivers, but low perceived need is also an important factor hindering help-seeking for mental health problems. Prior research suggested that a lack of need-perception is one of the major causes of unmet mental health needs [6, 7]. In our study, 3.9% of the 880 respondents without a perceived need do have a clinical assessed need. However, considering the 66 respondents with a clinical assessed need who did not consult a professional for their mental health, half (51.5%) did not report perceiving a need for mental health care, so insufficient awareness of one’s own mental health needs plays an important role in this study as well.

A major advantage of the study is the public mental health perspective. Other strengths of the study are the use of a representative probability sample and the inclusive nature of the study. For example, online participation was possible in six languages including Arabic, and the wider age range allowed 15 to 80-years old to participate.

It must be noted that the data collection took place between May and August 2021, which means some covid-19 related freedom-restrictions were still implemented and may have influenced the findings. Prior research showed no statistical difference between met and unmet need for mental health care, but point estimates were suggestive of higher unmet needs among those with a current mental disorder after the lock-down period [41]. Comparison with the province of Antwerp in the Belgian Health Interview survey suggests that the prevalence of mental health problems has risen substantially since 2018: the prevalence of depressive symptoms rose from 6 to 10%, anxiety disorder symptoms remained the same (11%), and alcohol abuse (based on the CAGE questionnaire only) doubled from 6 to 13% [42, 43]. However, no comparable Antwerp data are available on perceived or unmet needs.

Additionally, though validated instruments were used, the exclusive use of symptom screening questionnaires may be considered a limitation. These measures are indicative of mental disorders but tend to overestimate the true prevalence in the population [44, 45]. The dysfunction criterion was therefore added, leading to a more rigorous operationalization of clinically relevant mental health needs. However, this may have led to a higher proportion of false negatives. The regression analyses were also performed without dysfunction criterion, and the conclusions remained largely the same. The disorder type, comorbidity and severity were not considered when studying unmet needs. This may be relevant, as previous research suggests that men may be more likely than women to delay using health care for minor mental health concerns, but that gender effects diminish when problems are more serious [46]. Also, people with a substance use disorder tend to be less likely to perceive a need for care and seek treatment [47, 48].

As a final remark, we outline that only one quarter of the invited sample participated, despite two postal invitations and the possibility to participate online and offline. However, this response-rate was anticipated and the data were weighted to match the population distribution, also correcting for minor inequalities in non-response across strata. The forced response implementation may have caused some drop-out or reactance, although the “I don’t know” options prevented forced choice.

An important finding is that unmet mental health needs are high, with a population prevalence of 14.0% and 5.5% for perceived and clinical unmet needs, respectively. It should be noted that without the dysfunction criterion that was added to the operationalization of clinical mental health needs, the population prevalence of clinical unmet needs would be 14% as well (analysis available on request). This is higher than most estimates reported elsewhere, but different definitions and operationalizations complicate comparisons [2, 4]. However, the overlap between perceived and clinical unmet needs is small and are explained by different factors. Especially, more women perceived an unmet need for mental health care, but more men with a probable mental disorder did not seek care. Despite the higher prevalence of mental health problems in urban areas and among the less educated, little differences were found in unmet needs as regards to education and urbanicity. It must be noted that the relation between clinically assessed and perceived (unmet) needs and its associated factors is not fully addressed in this paper. Especially, further research should assess which people have both an assessed and perceived need, which people have a perceived or assessed need only, and in which way these subgroups differ. A combination of both approaches allows researchers and policymakers to assess the (unmet) need for mental health care on a population level with special attention to the individual perspective. Need perception is more related to help seeking, while assessed needs are more standardized and ‘objective’, although some degree of subjectivity is inevitably present to some degree in symptom scales as well.

Further efforts should be made to make mental health care more accessible for everyone. Insights in the barriers to care can lead to more targeted interventions in guiding people with mental problems to mental health care. Information and awareness campaigns are important to ensure that people recognize their own mental health needs and feel more confident and motivated to seek professional care. Familiarity with mental health services needs to be addressed, given that a lack of trust is a common barrier. Financial accessibility remains important, not only for seeking care, but also for obtaining sufficient care. To ensure that every individual with a mental health need receives adequate care, stepped care principles should be respected such that people with mild needs are helped in generalist or primary care services, and people with more severe needs in specialist services. Based on insights into the prevalence and distribution of unmet mental needs in the general population, a targeted health policy can be implemented, focusing on individuals with the highest (unmet) need. The nature of the present study where we collected data in particular regions, allows mental health services in the region to better tailor their care programs to the local needs. We believe that our findings, which apply to the general population in Antwerp, can to some degree be translated to other regions with a similar population structure and mental health care system, especially in Western-Europe. This is especially true for the associated factors of (unmet) mental health needs. Finally, the totality of health and social care needs of people with mental health problems should be addressed as well, so that not only the ‘treatment gap’ but the whole mental health ‘care gap’ can be reduced [3].

## Conclusions

This cross-sectional survey study evaluated common mental health needs (depression, anxiety and alcohol problems) in a representative general population sample in Antwerp, Belgium. Both self-reported perceived unmet needs and clinically assessed mental health needs measured by validated symptom screening scales were examined. One in five had a positive screening on one of the scales, but a dysfunction criterion was added to ensure clinical relevance, leading to a prevalence of 10.4% mental health needs in the population. One in six participants discussed their mental health with a professional in the past year. Among those with a mental health problem, about half (47.6%) had contact with a health professional for their mental health. In the general population, 5.5% had a clinically assessed unmet mental health need. More men, older people, and people without financial distress had an unmet mental health need.

With regard to perceived unmet needs, one in ten people thought they needed some help for their mental health but did not seek any, and 4.0% received some care but thought this was insufficient, resulting in a total population prevalence of 14.0% perceived unmet needs. As opposed to clinically assessed unmet needs, perceived unmet needs were more common among women, younger people, people with financial distress or a non-European background, and those with a mental health problem. Motivational and attitudinal barriers, especially the preference to handle problems on their own, are generally most often endorsed. However, cost is a main barrier to obtaining extra help.

## Data Availability

The datasets generated and/or analysed during the current study are not publicly available due to privacy regulations but are available from the corresponding author on reasonable request.

## Abbreviations

ESEMeD: European Study of the Epidemiology of Mental Disorders
PNCQ: Perceived Need for Care Questionnaire
PCZ: Primary care zone
UOR: Unadjusted odds ratio
AOR: Adjusted odds ratio
CI: Confidence interval
